# Curcumin increases exosomal TCF21 thus suppressing exosome-induced lung cancer

**DOI:** 10.18632/oncotarget.13499

**Published:** 2016-11-22

**Authors:** Hao Wu, Jingcheng Zhou, Chao Zeng, Da Wu, Zhimin Mu, Baokun Chen, Yuancai Xie, Yiwang Ye, Jixian Liu

**Affiliations:** ^1^ Department of Thoracic Surgery, Peking University Shenzhen Hospital, Shenzhen 518036, Guangdong Province, China

**Keywords:** curcumin, TCF21, DNMT1, exosome, lung cancer

## Abstract

Curcumin is a novel drug for lung cancer treatment. However, the mechanism underlying the anti-tumor effect of curcumin remains elusive. Previous evidences indicated that, the methylating transferase DNMT1 is downregulated by curcumin, and the transcription factor 21 (TCF21) is suppressed by DNMT1. We hereby attempt to elucidate the correlation between curcumin treatment and TCF21 expression. Exosomes derived from curcumin-pretreated H1299 cells were used to treat BEAS-2B cells, which induced proliferation, colony formation and migration of BEAS-2B cells. An increase in TCF21 expression in response to curcumin was also seen, as revealed by real-time PCR (RT-PCR) and western blot. Analysis using the GEO database (access #GSE21210) indicated that a positive correlation existed between TCF21 levels and lung cancer patient survival. TCF21 overexpression and knockdown was introduced to H1299 cells through lentiviral system, which led to suppression and promotion of tumor growth, respectively. We also demonstrated that DNMT1 expression was downregulated by curcumin. Therefore, curcumin exerts its anti-cancer function by downregulating DNMT1, thereby upregulating TCF21.

## INTRODUCTION

Lung cancer is the leading cause of cancer-related death worldwide and constitutes a major public health problem [1]. Effective therapy against lung cancer is of paramount importance to improve prognosis and survival of lung cancer patients. Resistance to existing chemotherapy drugs poses a serious challenge to lung cancer therapy, necessitating the development of new drugs against lung cancer. To date, a number of plant-derived anti-cancer compounds have been discovered [2]. One of them is curcumin, also known as diferuloylmethane, which is a phenolic compound isolated from *Curcuma longa* [3, 4]. Curcumin is well tolerated in high doses, and has been found to exert anti-inflammatory and anti-oxidative effects. However, the precise mechanism underlying the action of this drug in cancer is still unclear.

The anti-tumor role of curcumin is associated with DNA hypomethylation, which epigenetically controls gene transcription and genome stability [5–7]. Curcumin abrogates the catalytic activity of a methyltransferase, DNMT1, thereby affecting the methylation of a number of cancer-related genes [8–11]. TCF21, which can be inhibited by methylation, promotes mesenchymal to epithelial transition (MET) during organogenesis [12]. The reversal of MET, epithelial-to-mesenchymal transition (EMT), is a primary driver for cancer progression. Thus, the loss or reduced expression of TCF21 has been reported as a signature for malignant cancers [13, 14]. However, studies on TCF21 as a biomarker in lung cancer are still rare. Considering the demethylating activity of curcumin, there is possibility that the anti-tumor function of curcumin resides in the upregulation of TCF21.

Herein, we aimed to investigate the inhibitory role of curcumin in lung cancer, with particular interest in the link between curcumin and TCF21 expression. Here we focused on exosomes, which serve as delivery vehicles for miRNAs, anti-inflammatory agents, neural transmitters, etc. Delivered and released by exosomes, curcumin exerts an enhanced anti-cancer potency due to increased curcumin concentrations in recipient cells [15]. Furthermore, curcumin may also impair the tumor-promoting function of tumor-derived exosomes. Therefore, instead of directly using curcumin, we isolated exosomes derived from curcumin-pretreated lung cancer cells, to study curcumin's anti-cancer effect. To our knowledge, it is the first time that curcumin is shown to exert anti-cancer effects by upregulating TCF21. We also show that downregulation of TCF21 is associated with poor survival of lung cancer patients. On the other hand, overexpressing TCF21 inhibits tumor growth in a H1299 mouse tumor model. Our results may unravel the mechanism of curcumin action in lung cancer, and give rise to new diagnostic and therapeutic strategies for lung cancer.

## RESULTS

### Exosomes from curcumin-treated H1299 cells possess anti-tumor activity

**Figure 1:**
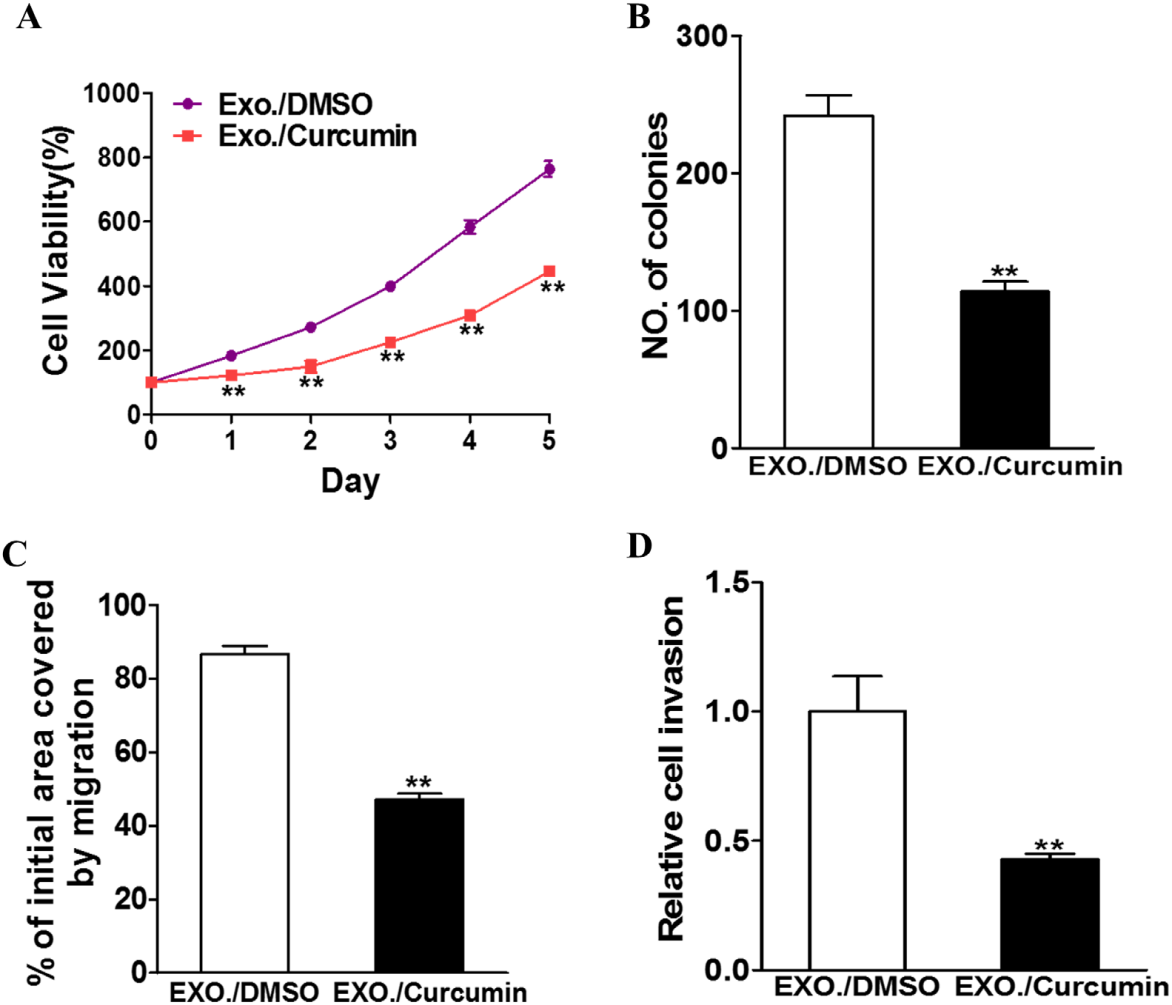
Curcumin treated exosome inhibit the exosomal development of lung cancer H1299 cells were treated with 10 μM curcumin or DMSO and after 48h the exosomes were collected from the supernatant of the cells. BEAS-2B cells were cultured with the exosomes respectively. **A**. CCK-8 assay was used to detect BEAS-2B cell viability. Data were presented as mean+SD from three independent experiments with triple replicates per experiment. ** p < 0.01. **B**. Cloning efficiency was measured by counting clone number growing in soft agar. ** p < 0.01. **C**. Migration rate was measured by wound healing assay. ** p<0.01. **D**. Invasion ability was measured by transwell invasion assay. ** p<0.01.

To specifically study the anti-tumor activity of curcumin-enriched exosomes in lung cancer cells, we isolated exosomes from H1299 cells that were cultured in medium containing 10 μM curcumin for 48 h. This generated exosomes containing high concentrations of curcumin. BEAS-2B cells were then incubated with these exosomes, followed by monitoring changes in cell proliferation, colony formation, migration and invasion. As a control, BEAS-2B cells were also incubated with exosomes generated from DMSO-treated H1299 cells. As shown in Figure 1, exosome-containing curcumin clearly induced a decrease in cell viability (Figure 1A, P<0.01), colony formation (Figure 1B, P<0.01), migration and invasion (Figure 1C and 1D).

### Curcumin upregulates exosomal TCF21 in lung cancer cells

**Figure 2:**
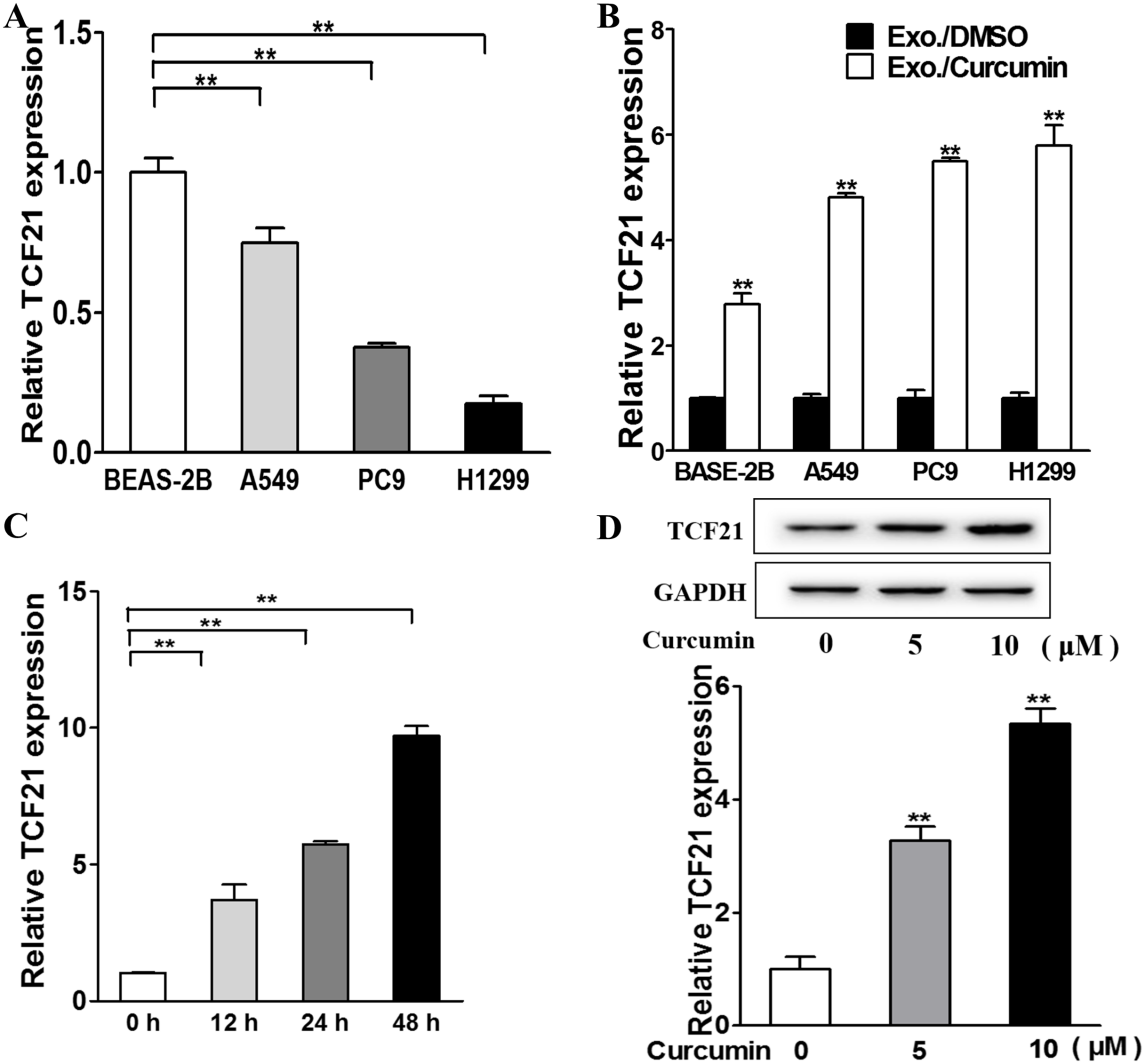
Exosomal TCF21 are down-regulated in lung cancer cell and are up-regulated by curcumin **A**. Relative exosomal TCF21 expression levels of normal lung cell and lung cancer cells were analyzed by RT-PCR and normalized to BEAS-2B group. ** p<0.01. **B**. The cells were treated with 10 μM curcumin or DMSO and after 48h the exosomes were collected from the supernatant of the cells. The relative exosomal TCF21 expression levels were analyzed by RT-PCR and normalized to DMSO group. ** p<0.01. **C**. The H1299 cells were treated with 10 μM curcumin or DMSO for different time points and the relative exosomal TCF21 expression levels were analyzed by RT-PCR and normalized to control group. ** p<0.01. **D**. H1299 cells were cultured with different concentration (0, 5, 10 μM) of curcumin and the exosomes were collected after 48 h. The BEAS-2B cells were cultured with the exosomes respectively and after 48h total proteins of each cell were subjected to western blotting and detected for TCF21 expression levels.

We next examined the correlation between exosomal TCF21 levels and curcumin. RT-PCR analysis indicated that downregulation of TCF21 was a characteristic of aggressive lung cancer, and the TCF21 levels were inversely correlated with the aggressiveness of cancer cells (aggressiveness: BEAS-2B < A549 < PC9 < H1299) (Figure 2A). Despite the difference in TCF21 basal expression, curcumin-containing exosomes universally upregulated TCF21 expression in these cells (Figure 2B). In H1299 cells, the TCF21 expression was upregulated with increasing duration of Exo./curcumin incubation (Figure 2C), as well as with the increasing concentration of curcumin (Figure 2D). Collectively, these data indicated that TCF21 upregulation was correlated with Exo./curcumin incubation. The role of curcumin in upregulating TCF21 was further validated *in vivo*. Tumors were initiated by injecting H1299 cells subcutaneously into nude mice, which then received either vehicle treatment or 300 mg/kg curcumin daily for 24 days. As shown in Supplementary Figure S1, TCF21 expression in curcumin-treated mice was significantly higher than those treated with vehicle, which again verified that curcumin was able to upregulate TCF21 expression.

### TCF21 is a positive biomarker in lung cancer prognosis

**Figure 3:**
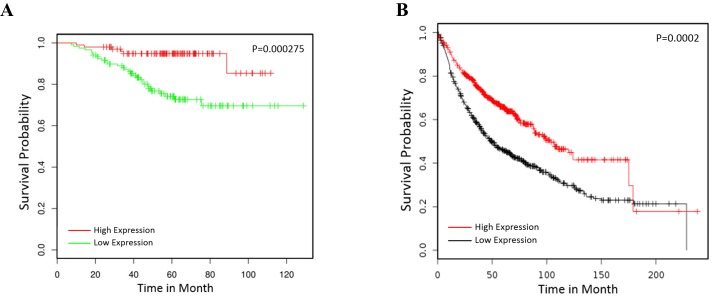
TCF21 is a positive biomarker in lung cancer prognosis **A**. The GEO database GSE31210 was employed to analyze the prognosis of lung cancer patients with high or low expression of TCF21 (TCF21 low expression group, n=120; TCF21 high expression group, n=102). **B**. The Kaplan Meier plotter was employed to analyze the prognosis of lung cancer patients by TCF21 (TCF21 low expression group, n=572; TCF21 high expression group, n=573).

To confirm that TCF21 is a prognostic biomarker in lung cancer patients, we examined the NCBI GEO database (access #GSE31210), which contains a large number of expression profiles of lung cancer patients, in search for correlation between TCF21 and patient survival. Clearly, low TCF21 expression is associated with poor survival of lung cancer (Figure 3A). The Kaplan-Meier plot indicated the same trend (Figure 3B), suggesting that TCF21 is an effective biomarker of patient survival.

### TCF21 coordinates with curcumin to suppress lung cancer

**Figure 4:**
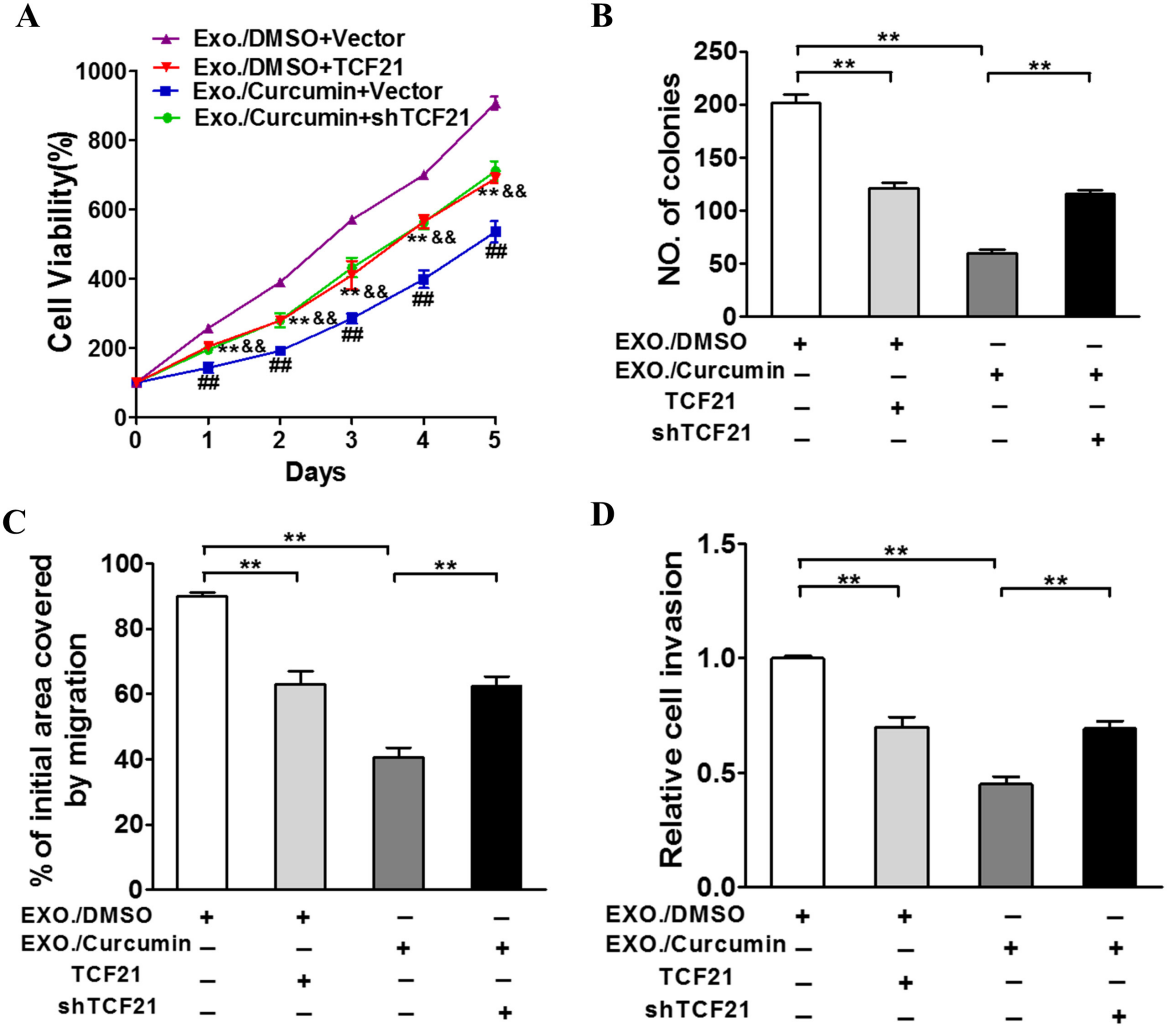
TCF21 participates in curcumin induced suppression role of exosomal lung cancer development H1299 cells were treated with 10 μM curcumin or DMSO and after 48h the exosomes were collected from the supernatant of the cells. BEAS-2B cells were culture with the exosomes respectively and were transfected with TCF21 or shTCF21 and Vector as control. **A**. CCK-8 assay was used to detect BEAS-2B cell viability. **B**. Cloning efficiency was measured by counting clone number growing in soft agar. **C**. Migration rate was measured by wound healing assay. **D**. Invasion ability was measured by transwell invasion assay. **: p<0.01 for comparison to Exo./DMSO+Vector group. &&: p<0.01 for comparison to Exo./Curcumin+Vector group. ##: p<0.01 for comparison to Exo./Curcumin+Vector group.

Provided that TCF21 downregulation is an indicator of malignant lung cancer, we next investigated how TCF21 overexpression and knockdown would alter the growth of cancer cells. TCF21-expressing plasmid was transfected into H1299 cells through lentivirus to induced TCF21 overexpression, and shTCF21 was introduced using Lipofectamine 2000. These studies were performed in the presence and absence of Exo./curcumin, respectively. As expected, curcumin and TCF21 individually exerted similar inhibitory effect on these cancer cells (Figure 4). Exo./cucumin and shTCF21 synergistically exerted inhibitory effects on cancer cells, resulting the lowest proliferation, colony formation and migration among all experimental groups (Figure 4). Furthermore, we tested if TCF21 knockdown induced curcumin resistance *in vivo*. As indicated in Supplementary Figure S2, a substantially higher tumor growth rate was observed in mice that received both shTCF21 and curcumin, compared to mice that received only curcumin, which was consistent with the positive correlation between TCF21 expression and patient survival.

### Downregulation of DNMT1 by curcumin is essential for the upregulation of TCF21

**Figure 5:**
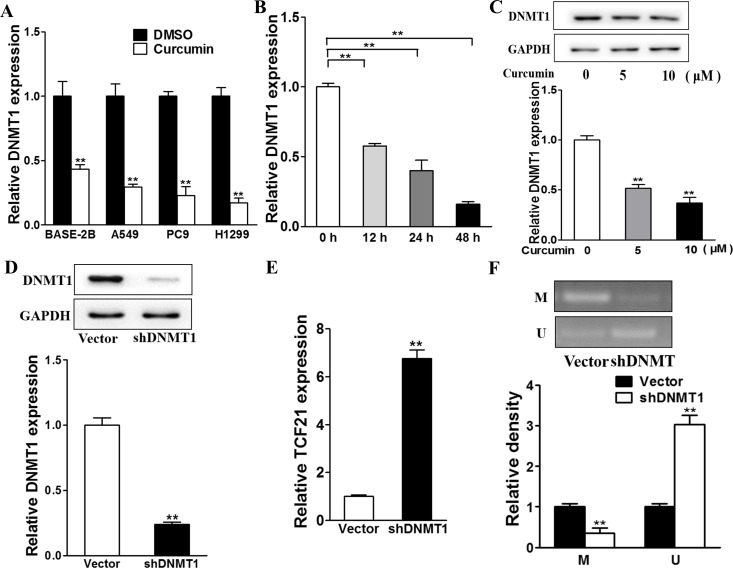
TCF21 is modified by DNA methylation transferase DNMT1 downregulated by curcumin **A**. The cells were treated with 10 μM curcumin or DMSO and after 48h the relative DNMT expression levels were analyzed by RT-PCR, which was normalized to DMSO group. ** p<0.01. **B**. The H1299 cells were treated with 10 μM curcumin or DMSO for different time points and the relative DNMT1 expression levels were analyzed by RT-PCR. ** p<0.01. **C**. H1299 cells were cultured with different concentration (0, 5, 10 μM) of curcumin and after 48h total proteins were detected for DNMT1 expression levels using western blotting analysis. **D**. The H1299 cells were transfected with shDNMT1 or Vector for 48h. Western blotting analysis was used to detect DNMT expression levels. **E**. The cells were transfected with shDNMT1 or Vector and after 48h the exosomes were collected from the supernatant of the cells. Relative exosomal TCF21 expression levels were analyzed by RT-PCR. ** p<0.01. **F**. The H1299 cells were transfected with shDNMT1 or Vector and the MSP assay were performed to detect the methylation levels of TCF21 promotor. ** p<0.01.

We next investigated whether DNMT1 expression was regulated by curcumin. As shown in Figure 5A, curcumin was able to downregulate DNMT1 expression in BEAS-2B, A549, PC9, and H1299 cells. This downregulation was enhanced with longer duration of curcumin treatment (Figure 5B). Western blot analysis indicated that higher concentration of curcumin further lowered the DNMT1 levels (Figure 5C). Thus, DNMT1 downregulation was mediated by curcumin, in time- and dose-dependent manners. To confirm the correlation between TCF21 and DNMT1, we performed DNMT1 knockdown, which led to downregulation of DNMT1 protein expression (Figure 5D) and an over 6-fold upregulation of TCF21 mRNA (P<0.01; Figure 5E). Methylation-specific PCR was also performed to examined the methylation levels of TCF21 promoters. Transfection of shDNMT1 induced a downregulation of methylated TCF21 promoter, as opposed to the upregulation of unmethylated TCF21 promoter, which is in accordance to the methylation function of DNMT1 (Figure 5F). Collectively, these data supported the causal relationship between curcumin regulation and DNMT1 methylation of TCF21.

### Tumor suppressing role of TCF21

**Figure 6:**
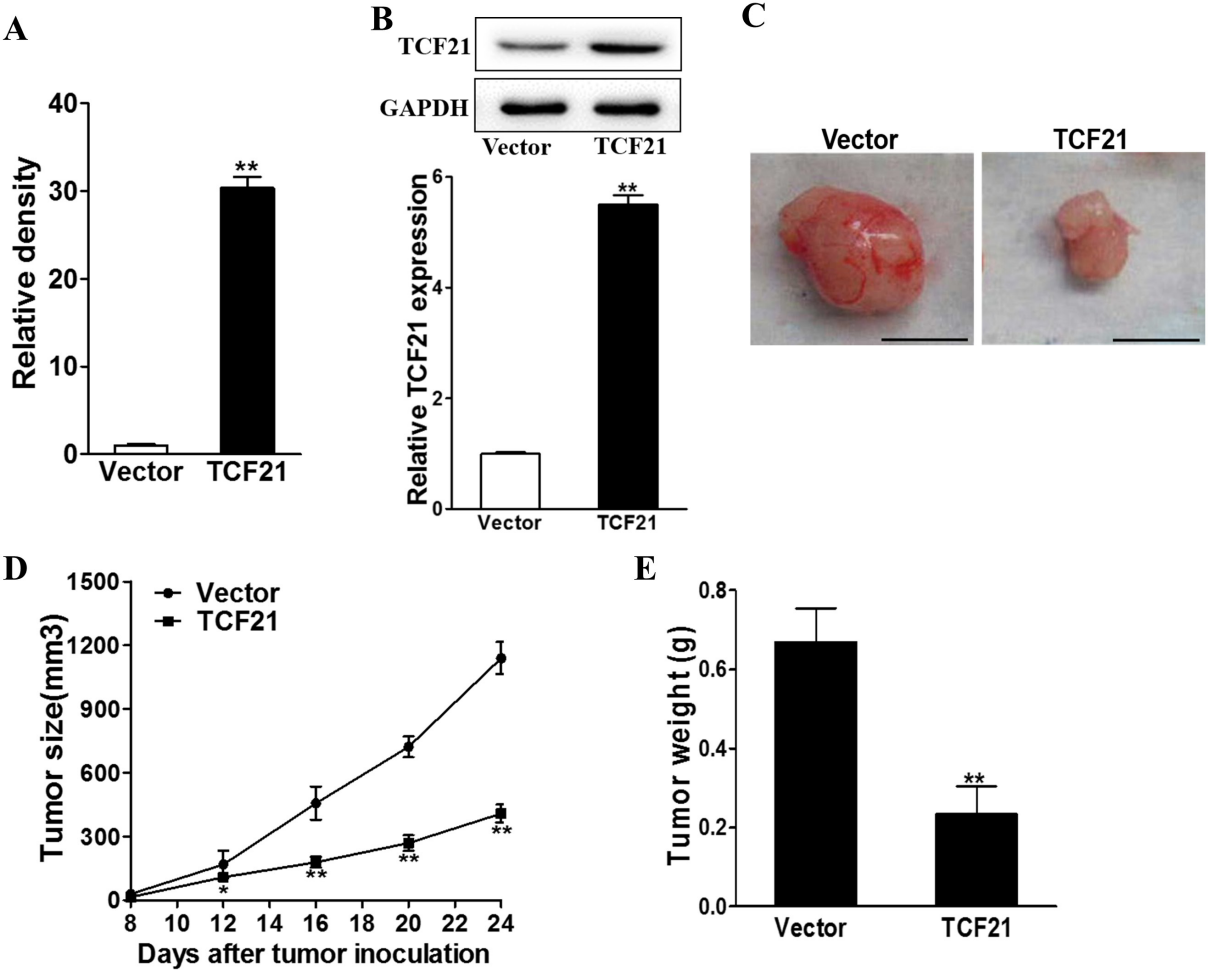
TCF21 suppresses tumor growth The H1299 cells were infected with TCF21 or Vector lentivirus to establish stable cell lines. **A-B**. Relative TCF21 expression levels of the stable cells were analyzed by RT-PCR and western blotting. ** p<0.01. **C, D, E**. H1299/Vector and H1299/TCF21 cells were dispersed in 100 μl of serum-free 1640 medium and were subcutaneously injected into each side of posterior flank of the nude mice (n=4). Tumors were measured every four days since they were apparently seen and the volumes were calculated using the following formula: volume = 0.5 × Length × Width^2^. The tumor was excised and weighed after 24 days with representative pictures of tumors shown (Bar = 10 mm).

To explore the inhibitory role of TCF21 *in vivo*, we established TCF21 overexpressing H1299 cell lines through lentivirus transfection of TCF21 expressing plasmid. RT-PCR revealed that the cells transfected with TCF21 expressing plasmid possessed approximately 30 times higher TCF21 level than those transfected with lentivirus vector (Figure 6A). After inoculation, we observed that cells with TCF21 overexpression showed a much lower growth rate than those expressing lentivirus vector (P<0.01), as evident by smaller tumor size (Figure 6B and 6C) and lower tumor weight (Figure 6D). All these data pointed to the conclusion that TCF21 greatly reduced tumor growth in a mouse model.

## DISCUSSION

In the present study, we showed that exosomes isolated from curcumin-pretreated H1299 cells exhibited anti-tumor activity. These findings are consistent with the notion that chemotherapy drugs may exert their anti-cancer effects via exosomes. Tumor-derived exosomes, in most cases, take pro-tumorigenic roles in cancer by exhibiting immunosuppressive properties and transporting RNAs/proteins; it may also interfere with cytotoxic effect of chemotherapy drugs through drug exportation [16]. Nevertheless, exosomes can also act as treatment response markers since effective cancer therapy is possibly facilitated by reducing exosome secretion and/or inhibiting exosome functions [17]. In fact, removal of exosomes has been proposed as an effective therapeutic strategy for cancers [18]. It is also worth noting that tumor-derived exosomes encapsulate curcumin. As a result, curcumin is transported to recipient cells at a high local concentration, thereby enhancing its anti-cancer effects. These curcumin-carrying exosomes distribute curcumin in blood circulation, and protect curcumin from biodegradation, improving the *in vivo* bioavailability. Inspired by this, exosomes have been developed as a curcumin delivery system to exert anti-inflammatory effects [15]. Besides, exosomes were also used for delivering siRNA, miRNA, DNA and drugs [19, 20]. These naturally occurring, endogenous nanoparticles are advantageous over artificial nanoparticles in minimized antigenicity, enhanced stability and higher safety.

We confirmed that TCF21 is a biomarker for lung cancer. Previously, downregulation of TCF21 was demonstrated to be associated with poor survival in clear cell renal cell carcinoma [13], urological cancer [21], head and neck cancer [22], gastric cancer [23] and lung cancer [24]. Here, we validated the diagnostic value of TCF21 in lung cancer by showing that TCF21 downregulation is a characteristic of lung cancer patients with poor survival. Accordingly, TCF21 overexpression and suppression had pro-tumorigenic and tumor-suppressive effects, respectively. Importantly, Exo./curcumin treatment upregulated TCF21, suggesting that the TCF21 is involved in the anti-cancer effect of curcumin. TCF21 downregulation is associated with EMT, a process that generates cancer stem cells (CSC) and promotes cancer cell migration, invasion and metastasis. By promoting TCF21 expression, curcumin impairs tumor progression by inhibiting the emergence of cancer stem cells, thereby containing the cancer dissemination. Moreover, it is widely accepted that EMT is involved in cancer resistance [25]. It is possible that TCF21 downregulation is associated with the drug resistance of lung cancer. Thus, detection of the methylated TCF21 levels is a promising approach to determine lung cancer risk, in order to improve the clinical management of lung cancer. In addition, upregulating TCF21 expression is a viable strategy for suppressing lung cancer progression. We showed that H1299 cells overexpressing TCF21 exhibited a slower tumor growth than normal H1299 *in vivo*. In line with this result, we speculated that delivery of TCF21-expressing plasmids intravenously to tumor tissue would also exert tumor inhibitory effects. Indeed, various early studies have reported that DNA-delivery systems have enabled overexpression or knockdown of cancer-specific genes to suppress tumor growth [26–28]. Since TCF21 is constitutively highly expressed in normal cells, this strategy is superior than conventional chemotherapy, in that it would not cause toxicity to normal tissues. Furthermore, TCF21-based gene therapy can be combined with curcumin treatment to synergistically maximize the therapeutic effects.

As promoter methylation is the primary mechanism of TCF21 regulation, and curcumin covalently binds to DNMT1, we sought to validate the link between DNMT1 and TCF21. First, we recapitulated that DNMT1 was downregulated by curcumin treatment; we then showed that knockdown of DNMT1 upregulated TCF21 expression. In this regard, silencing DNMT1 with shRNA is another approach to inhibit tumors. This result is consistent with previous report that aberrant methylation was associated with enhanced lung cancer progression [29].

In summary, we hereby report that TCF21 is a putative diagnostic and therapeutic target in lung cancer. Furthermore, we showed that curcumin downregulated DNMT1 to promote TCF21 expression. Our findings could support further development of diagnostic and therapeutic approaches for lung cancer based on curcumin.

## MATERIALS AND METHODS

### Materials

Cells used in this study, including BEAS-2B, A549, PC9 and H1299 were acquired from American Type Culture Collection (ATCC, Rockville, MD, USA), and cultured in RPMI-1640 medium supplemented with 10% fetal bovine serum (FBS; Invitrogen, Carlsbad, CA, USA) and 1% Penicillin Streptomycin (Pen/Step) (Invitrogen). Curcumin (70% purity) was acquired from Fluka (Sleelze, Germany). Cells were cultured in an incubator maintained at 37°C with 5% CO_2_.

### Curcumin treatment and exosomes extraction

Curcumin powder was dissolved and added to culture medium at the concentration of 10 μM for different durations, ranging from 24 to 72 h. This concentration and treatment duration was chosen based on a previous protocol [30]. For dose-dependent study, cells were also exposed to 5 μM study for 24 h. As a control, cells were treated with DMSO. Cells were grown until reaching 90% confluency, and washed with PBS, followed by incubating with fresh complete media free of exosomes supplemented with FBS for 2 days. Briefly, isolation of exosomes were performed by differential centrifugation at 4°C in conditioned medium, first at 300 *g* for 10 min, then at 2,000 *g* for 20 min, followed by filtration with a 220 μm filter for cell debris removal. The exosomes were then pelleted at 4°C by centrifugation at 100,000 *g* for 90 min. Pellets were re-suspended in PBS and centrifuged again at 100,000 *g* for another 90 min. Measurements of the size and concentration of the exosomes were performed using qNano (Izon Science, New Zealand), which were then ready for RNA or protein extraction.

### Cell viability, colony formation, and migration assays

Cell counting kit 8 (CCK-8) assay was used to assess cell viability. Cells were first seeded in 96-well plates. CCK-8 solution was added to each well followed by incubation for 2 h. Cell viability was measured based on the absorbance at 450 nm. To evaluate colony-forming ability of cells, cells were firstly trypsinized and the resulted solution was passed through cell strainer to produce single-cell suspensions. Then cells were plated in 6-well plates coated with soft agar at the density of 50 cm^-12^. Two milliliters of culture medium was added into each well. Cells were cultured for 3 days before fixed with staining with 6% glutaraldehyde and 0.5% crystal violet. Plates were subsequently allowed to dry with air at room temperature. Colony counting was performed using a stereomicroscope. Migratory ability of cells were evaluated with scratch wound assay and transwell matrigel invasion assay. For scratch wound assay, cells at the confluency of over 80% were subjected to scratch with a 10 μL pipette tip. The gap areas immediately after gaps were made and at 24 h after scratching were documented by capturing images of the gap using a microscope. Migratory ability was correlated with the rate of cells refilling the gap areas. For transwell matrigel invasion assay, cells were plated in the upper chamber of the transwell apparatus (Corning Costar, Corning, NY), the bottom of which was coated with matrigel. Cells that migrated at the lower chamber were fixed. MTT containing medium was added to the cells, and incubated for 4 hours. Subsequently, medium was removed and DMSO was added to dissolve the formazon crystal formed in cells. Measurement of absorbance at 570 nm was used to calculate the number of cells.

### RT-PCR and western blot

Cells cultured to approximately 80% confluency in 6-well plates were harvested by trypsinization. Extraction of total RNA was performed with the TRIzol^®^ Reagents extraction kit (Thermo Fisher, San Jose, CA, USA). Synthesis of cDNA was performed using the Maxima H Minus First Strand cDNA Synthesis Kit (Thermo Fisher). Real-time PCR was performed using the MasterCycler RealPlex 4 from Eppendorf (Hamburg, Germany). The SYBR Green Master Mix was used for PCR reaction. Quantification of mRNA level was using the 2^-ΔΔT^ method.

For western blot, cells were lysed with T-PER Tissue Protein Extract Reagent (Thermo Fisher Scientific), supplemented with Proteinase Inhibitor Cocktail (Invitrogen) and phenylmethylsulfonyl fluoride (PMSF, Sigma-Aldrich, Saint Louis, MO). Protein lysates of 30 μg was loaded into Mini-PROTEAN^®^ Precast Gel (4-20%), which was used for SDS-PAGE using the Tetra Vertical Electrophoresis Cell (Biorad). Resolved proteins were transferred to nitrocellulose membranes and incubated with antibodies according to standard procedures. Antibodies used in this study included: rabbit anit-TCF21 (ab32981, Abcam), rabbit anti-GAPDH (ab37168, Abcam), and HRP-conjugated goat anti-rabbit (ab6721, Abcam).

### GEO database analysis

The Gene Expression Omnibus (GEO) database from the National Center for Biotechnology Information (NCBI) was used to confirm that TCF21 downregulation is a signature for lung cancer patients with a poor survival. The dataset with access number of GSE21210, which contains a larger cohort of lung cancer patients (n=102), was used for data analysis.

### Lentivirus-mediated gene overexpression and RNA silencing

TCF21 overexpression and knockdown was performed based on a modified protocol described previously [31]. Briefly, to establish the TCF21-expressing cell line, the cDNA encoding TCF21 (OriGene #SC12508) was cloned into the pWPI lentiviral backbone (#12254, Addgene, Inc. Cambridge, MA) to construct the transfer plasmid. Oligonucleotides encoding shTCF21 [31] and shDNMT1 [32] were inserted to the same plasmid. HEK293T cells (1 × 10^6^) were seeded into tissue culture dish until 50% confluence. Transfection was done in DMEM medium, co-transfecting with packaging vectors and Mission Lentiviral packaging mix (Sigma-Aldrich) and Lipofectamine 2000 agent (Invitrogen). Virons that produced at 24 h-incubation were purified with centrifugation at 3000 rpm and filtration through a 0.45 μm-pore-size membrane. These virons were used to transfect cells cultured in 6-well plates. Cells stably expressing TCF21, shTCF21, or shDNMT1 were selected at 48 h after transfection using 2 μg/mL puromycin.

### Methylation-specific PCR analysis

Methylation-specific PCR (MSP) for DNMT1 was performed based on a protocol reported previously [33]. Briefly, a pair of primers for methylated DNMT1 and unmethylated DNMT1 were used for PCR. Amplified DNA was used for gel electrophoresis and further quantification.

### Tumor models

Normal H1299 cells and H1299 cells that stably expressed TCF21 at 1 × 10^6^ were inoculated subcutaneously into Balb/c athymic mice. Tumor were measured every four days since they were palpable. Tumor volume was calculated using volume = 0.5 × length × width^2^. Tumors were harvested after 24 days, and tumor weight were measured and tumors were photographed using a digital camera. This study was carried out in strict accordance with the recommendations in the Guide for the Care and Use of Laboratory Animals of the National Institutes of Health. The protocol was approved by the Committee on the Ethics of Animal Experiments of Peking University Shenzhen Hospital. All surgery was performed under sodium pentobarbital anesthesia, and all efforts were made to minimize suffering.

### Statistical analysis

All experiments were performed in triplicates unless otherwise stated. Data were represented as mean±SD, and analyzed using Student's t-test. Differences between groups were considered statistically significant if p<0.05.

## CONCLUSION

The anti-cancer effects of curcumin are associated with upregulation of TCF21, mediated by downregulation of DNMT1. The results here extend our knowledge on the mechanism of curcumin in cancer treatment, and provide us with new biomarkers for advancing cancer diagnostic and therapeutic strategies.

## SUPPLEMENTARY FIGURES


