# Supplementary material for: Curcumin increases exosomal TCF21 thus suppressing exosome-induced lung cancer
Source: Oncotarget. 2016 Nov 22;7(52):87081–90. doi: 10.18632/oncotarget.13499 (PMC5349972; doi:10.18632/oncotarget.13499)
Supplement: Supplementary file 1 [file oncotarget-07-87081-s001.pdf]

Curcumin increases exosomal TCF21 thus suppressing exosome-induced lung cancer

SUPPLEMENTARY FIGURES

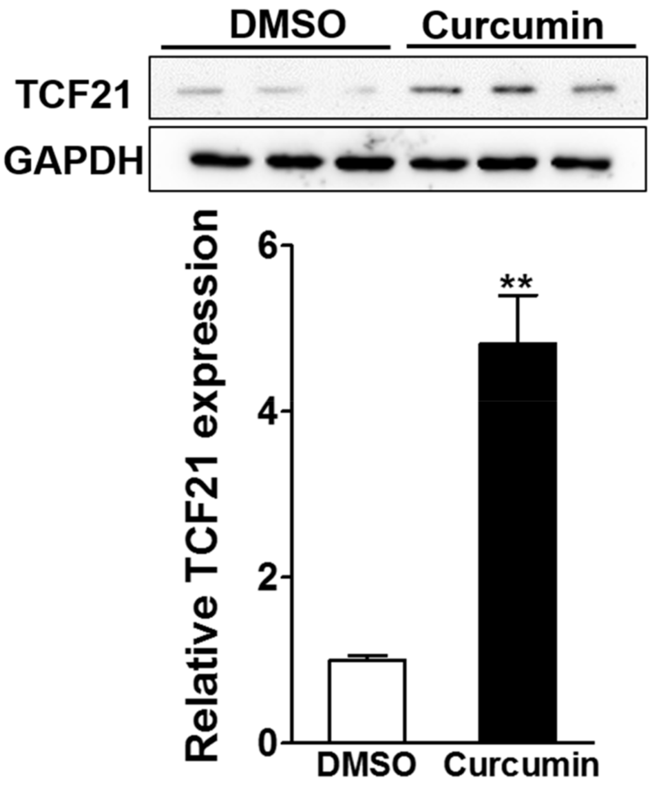

Supplementary Figure S1: Curcumin treatment also promotes TCF21 expression *in vivo*. A. H1299 cells were dispersed in 100 µl of serum-free 1640 medium and were subcutaneously injected into each side of posterior flank of the nude mice, then the mice were then divided into two groups to receive vehicle as control and 300 mg/kg curcumin (n=4). After 24 days, the tumors were excised and the total proteins of each tumor were subjected to western blotting and detected for TCF21 expression levels. ** p<0.01.

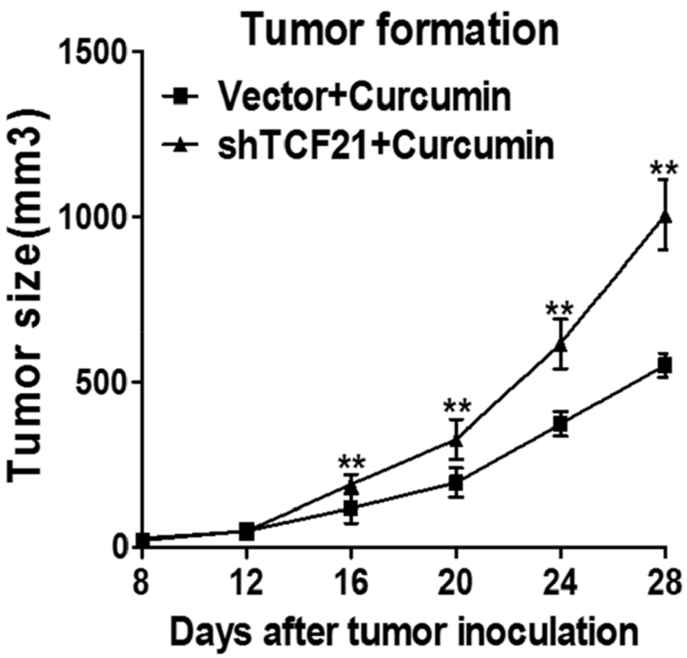

Supplementary Figure S2: TCF21 knockdown tumor cells line is resistance to curcumin treatment *in vivo*. The H1299 cells were infected with shTCF21 or Vector lentivirus to establish stable cell lines. **A.** H1299/Vector and H1299/shTCF21 cells were dispersed in 100 µl of serum-free 1640 medium and were subcutaneously injected into each side of posterior flank of the nude mice, then the mice were then divided into two groups to receive vehicle as control and 300 mg/kg curcumin (n=4). Tumors were measured every four days since they were apparently seen and the volumes were calculated using the following formula: volume = 0.5 × Length × Width^2.
